# Adrenal hemangioma: A rare incidental tumor managed with laparoscopic partial adrenalectomy

**DOI:** 10.1016/j.eucr.2025.103305

**Published:** 2025-12-07

**Authors:** Mehdi Dadpour, Nooshin Ahmadi, Mahyar Najarian, Niloofar Irandoost, Arash Ranjbar, Farzad Hadaegh

**Affiliations:** a Shahid Labbafinejad Medical Center, The Center of Excellence in Urology, Urology and Nephrology Research Center, Research Institute for Urology and Nephrology, Shahid Beheshti University of Medical Sciences, Tehran, Iran; b Research Institute for Endocrine Sciences, Shahid Beheshti University of Medical Sciences, Tehran, Iran; c Prevention of Metabolic Disorders Research Center, Research Institute for Metabolic and Obesity Disorders, Research Institute for Endocrine Sciences, Shahid Beheshti University of Medical Sciences, Tehran, Iran

**Keywords:** Adrenal hemangioma, Laparoscopy, Partial adrenalectomy, Incidentaloma

## Abstract

Adrenal hemangiomas are rare, non-functional vascular tumors often discovered incidentally and frequently mistaken for malignant adrenal masses. We present a 49-year-old man with an asymptomatic, hypervascular right adrenal lesion detected on routine imaging. Although hormonal evaluation was normal and the likelihood of malignancy was low, radiologic features could not definitively exclude a neoplasm. The patient underwent laparoscopic partial adrenalectomy with uneventful recovery. Histopathology confirmed adrenal hemangioma. This case highlights the diagnostic challenges of hypervascular adrenal masses and considerations for adrenal-sparing surgical management.

## 1 Introduction

With the increasing use of cross-sectional imaging such as computed tomography (CT) and magnetic resonance imaging (MRI), the detection of incidentally discovered adrenal masses—commonly termed adrenal incidentalomas—has risen markedly, with a reported prevalence of up to 5 % in the general population. These lesions, defined as adrenal masses greater than 1 cm detected during imaging for unrelated conditions, are usually benign, with adrenal cortical adenomas being the most frequent findings.^1^^,^^2^

Adrenal hemangiomas (AHs) are exceedingly rare benign vascular tumors that account for a minute fraction of adrenal incidentalomas. Since the first case was described in 1955, less than 100 cases have been reported in the literature.^3^ AHs are typically non-functional and asymptomatic, often identified incidentally. However, because of their rarity and non-specific imaging characteristics, they are frequently mistaken for other adrenal neoplasms, pheochromocytomas or adrenocortical carcinomas, leading to extensive diagnostic workup or unnecessary intervention. Radiologic features alone often cannot reliably differentiate benign from malignant adrenal lesions, and surgical excision remains the definitive method for diagnosis, especially for lesions larger than 4–6 cm or with atypical imaging findings.^3^^,^^4^

We present a case of a 49-year-old man with an incidentally detected right adrenal mass initially suspected to be malignant, which was histopathologically confirmed as a benign adrenal hemangioma following laparoscopic partial adrenalectomy.

## 2 Case report

**Fig. 1:**
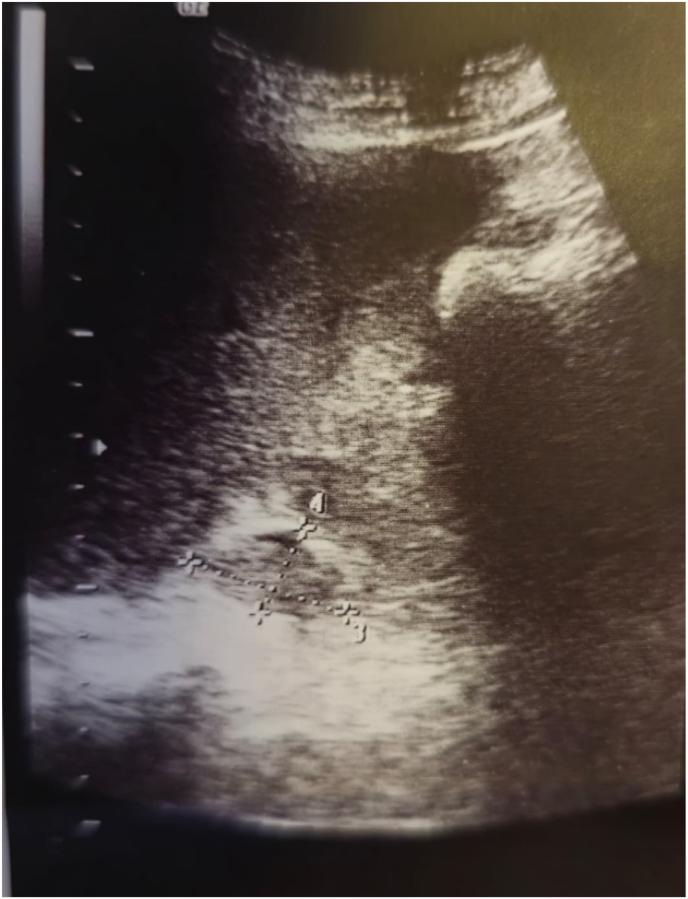
The ultrasound revealed a well-defined, solid hypoechoic mass in the right adrenal gland, measuring approximately 32 × 19 mm.

A 49-year-old man with no significant past medical history was referred to our urology clinic after a right adrenal lesion was identified during a routine abdominal ultrasonography performed solely for a general health check-up. He was completely asymptomatic, with no reports of abdominal discomfort, weight loss, hypertension, palpitations, headaches, or other endocrine-related symptoms. The ultrasound revealed a well-defined, solid hypoechoic mass in the right adrenal gland, measuring approximately 32 × 19 mm (Fig. 1), prompting a recommendation for advanced imaging with computed tomography (CT) for further characterization.

**Fig. 2:**
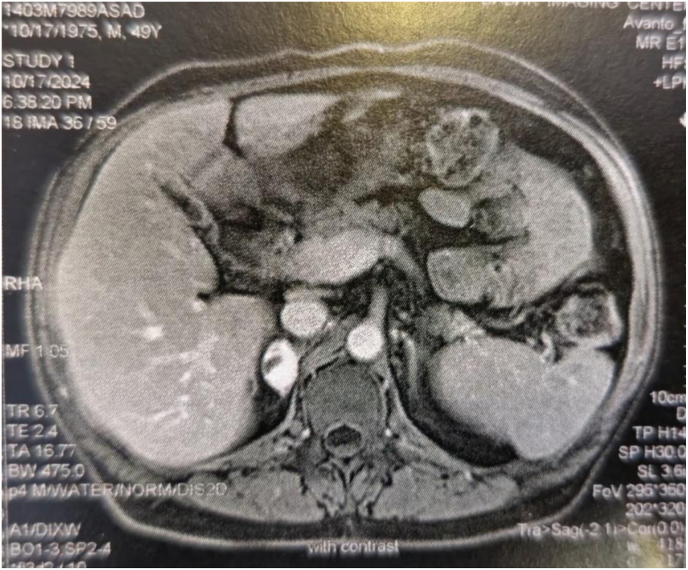
Contrast-enhanced CT demonstrated a hypervascular, heterogeneous mass in the right adrenal region without calcification, fat attenuation, or adjacent organ invasion.

Contrast-enhanced CT demonstrated a hypervascular, heterogeneous mass in the right adrenal region without calcification, fat attenuation, or adjacent organ invasion (Fig. 2). No evidence of metastatic disease or lymphadenopathy was observed. Given the hypervascular pattern—raising suspicion for pheochromocytoma or adrenocortical carcinoma—a complete hormonal workup was performed, including serum cortisol, 24-h urine metanephrines and nor-metanephrine, aldosterone, and renin levels. All biochemical results were within normal limits, consistent with a non-functional adrenal tumor.

Due to the vascular nature of the lesion, the inability to exclude malignancy radiologically and patient's concern, laparoscopic adrenalectomy was planned. The procedure was performed under general anesthesia with the patient in the left lateral decubitus position. The abdomen was accessed by an open technique. A 10-mm umbilical trocar was inserted for the laparoscope lens, followed by three 5-mm trocars for working ports.^5^ The right adrenal gland was carefully mobilized, and the mass was excised (adrenal sparing surgery) without intraoperative complications.^6^ Estimated blood loss was minimal.

Gross pathological assessment revealed a well-circumscribed vascular lesion measuring 2.2 × 1.1 × 1.0 cm^3^. Histologic examination showed numerous dilated vascular channels lined with endothelial cells. Immunohistochemistry (IHC) demonstrated strong CD31 and CD34 positivity within the vascular spaces, while calretinin positivity was restricted to the surrounding adrenal cortex. The Ki-67 proliferative index was low (3–4 %). These findings confirmed the diagnosis of adrenal hemangioma.

The patient recovered uneventfully and remained asymptomatic at follow-up, with no evidence of recurrence or adrenal insufficiency, normal postoperative hormonal evaluation, and complete return to daily activities.

## 3 Discussion

Adrenal hemangiomas are rare benign vascular tumors that constitute a very small percentage of adrenal incidentalomas. They are most commonly cavernous in nature and are typically non-functional. Because they lack specific symptoms and biochemical abnormalities, most are discovered incidentally during imaging performed for unrelated conditions. Their rarity, combined with nonspecific radiologic characteristics, makes preoperative diagnosis challenging.^4^

On contrast-enhanced CT or MRI, adrenal hemangiomas may present as hypervascular lesions with heterogeneous enhancement due to thrombosis, fibrosis, or calcification within the tumor. However, these features overlap with the radiologic findings of pheochromocytoma, adrenocortical carcinoma, or metastatic lesions.^7^ Therefore, distinguishing hemangiomas from malignant or functional adrenal tumors based solely on imaging is often not feasible. In our case, the hypervascular pattern prompted a malignancy workup and ultimately led to the decision for surgical removal.

Current management guidelines for adrenal incidentalomas recommend surgical resection for masses larger than 4–6 cm, those that exhibit interval growth, those that are hormonally active, or when malignant potential cannot be excluded.^7^ Although our patient's lesion was relatively small, its vascularity and imaging appearance raised enough concern to justify operative treatment. Laparoscopic adrenalectomy remains the preferred approach for benign adrenal tumors due to reduced morbidity and faster postoperative recovery.

Definitive diagnosis relies on histopathological evaluation, demonstrating dilated vascular channels lined by endothelial cells. Immunohistochemical markers such as CD31 and CD34 support a vascular origin, as seen in this patient.^8^ Prognosis after complete excision is excellent, with extremely low risk of recurrence or functional impairment.

This case emphasizes the diagnostic difficulty associated with rare adrenal hemangiomas and highlights the importance of considering them in the differential diagnosis of hypervascular adrenal masses. Early surgical intervention not only provides a definitive diagnosis but also alleviates patient anxiety regarding the possibility of malignancy. Although complete adrenalectomy remains the standard approach when malignancy cannot be excluded, partial adrenalectomy or adrenal-sparing surgery may be considered in select cases involving small, clearly benign-appearing, or hormonally active lesions in order to preserve adrenal function. However, due to the uncertain preoperative diagnosis and the vascular nature of hemangiomas, adrenal-sparing procedures must be approached cautiously and are typically reserved for carefully selected patients in whom the risk of malignancy is low. In our patient, the mass was relatively small and the biochemical evaluation was normal, suggesting a low probability of malignancy.

## 4 Conclusion

Adrenal hemangiomas are rare benign vascular tumors that often mimic malignant adrenal lesions on imaging. Surgical excision remains the most reliable method for definitive diagnosis when malignancy cannot be excluded. This case highlights the importance of considering adrenal hemangioma in the differential diagnosis of hypervascular adrenal masses to guide appropriate management.

## CRediT authorship contribution statement

**Mehdi Dadpour:** Writing – review & editing, Writing – original draft, Supervision, Project administration, Methodology, Investigation, Conceptualization. **Nooshin Ahmadi:** Methodology, Investigation, Formal analysis, Data curation, Conceptualization. **Mahyar Najarian:** Writing – original draft, Visualization. **Niloofar Irandoost:** Writing – original draft, Visualization. **Arash Ranjbar:** Methodology, Investigation. **Farzad Hadaegh:** Writing – review & editing, Supervision, Project administration, Investigation, Data curation, Conceptualization.

## Fund

No fund was used in this report.

## Conflict of interests

All authors declare that they have no conflict of interests.
